# A decade of insight into ExlA toxin research and the biotechnological potential of non-virulent *Pseudomonas aeruginosa* strains

**DOI:** 10.1128/jb.00496-25

**Published:** 2026-02-25

**Authors:** Selene García-Reyes

**Affiliations:** 1 Programa de Investigadoras e Investigadores por México-Secihti-Instituto de Química, Universidad Nacional Autónoma de México 7180, , Ciudad de México, México; Dartmouth College Geisel School of Medicine, Hanover, New Hampshire, USA

**Keywords:** *Pseudomonas*, toxin, pathogenesis, virulence, ExlA, biotechnological

## Abstract

*Pseudomonas aeruginosa* remains a critical global health concern because of its virulence and multidrug resistance. While clades 1 and 2 are well documented for utilizing the type 3 secretion system (T3SS) as a primary pathogenic mechanism, clade 5 strains have drawn attention for their reliance on alternative virulence factors, such as Exolysin A (ExlA). Over the past decade, research has provided deeper insights into ExlA, a pore-forming toxin associated with clades 3 and 5, offering a unique perspective on the pathogenic strategies of these less-virulent strains. This review explores the regulatory pathways of ExlA expression, its effects on host cells, and its role in the broader context of the reduced virulence of clade 5. By examining the potential biotechnological applications of clade 5 strains, this review highlights therapeutic opportunities presented by leveraging their less harmful nature. The synthesis of this knowledge contributes to a broader understanding of how non-T3SS-dependent mechanisms can be harnessed to develop innovative anti-virulence therapies and alternative biotechnological applications.

## INTRODUCTION

Antibiotic-resistant pathogens pose a significant threat to global health, complicating the treatment of infections and increasing the burden on healthcare systems. Among these pathogens, *Pseudomonas aeruginosa* is a major concern because of its ability to cause both acute and chronic infections in immunocompromised individuals (1–4), including those with cancer, HIV, diabetes, or cystic fibrosis (5, 6). This bacterium is responsible for many infections, including gastrointestinal, respiratory, ocular, urinary tract, skin, and bloodstream infections (7). *P. aeruginosa* is frequently identified as a cause of nosocomial pneumonia (8). The World Health Organization has classified carbapenem-resistant *P. aeruginosa* as a pathogen of high priority for investigation and an urgent need for new treatment alternatives (9).

The pathogenicity of *P. aeruginosa* is attributed to several virulence factors (VFs), including pyocyanin, elastase LasB, and rhamnolipids (mono-rhamnolipids and di-rhamnolipids) (10, 11). Additionally, these secondary metabolites can be used in various biotechnological applications, as explained later (12–14).

Another critical virulence mechanism that causes cytotoxicity is the type 3 secretion system (T3SS), a needle-like structure that injects toxins, such as ExoS, ExoU, ExoY, and ExoT, into host cells (15, 16). Since 2014, a subset of *P. aeruginosa* strains has been identified that lack the T3SS, but instead use a simpler secretion mechanism, known as the two-partner secretion (TPS) system, to secrete the cytotoxic ExlA toxin (17, 18). These T3SS-negative strains have been isolated from human infections, as well as from environmental sites, suggesting that ExlA represents an alternative pathogenic mechanism (17, 19). Although strains from *P. aeruginosa* clades 3 and 5 carry genes that encode this new virulence factor (20, 21), not all of them express it, resulting in attenuated or non-virulent phenotypes (17).

This review aims to compile and analyze studies related to ExlA toxin, with a focus on its regulation and mode of action in *P. aeruginosa*. We hypothesized that the ExlA toxin plays a pivotal role in the virulence of T3SS-negative strains and that a deeper understanding of its mechanisms could open the door to novel therapeutic strategies. Additionally, we explored the biotechnological applications of *P. aeruginosa* strains that possess genes for ExlA but do not express them and are thus non-virulent.

A clearer understanding of the role of ExlA in *P. aeruginosa* pathogenesis is critical for addressing the challenges posed by this pathogen, especially considering the rising antibiotic resistance. By unraveling the mechanisms of ExlA, we can deepen our understanding of bacterial virulence and enhance our approaches to combat *P. aeruginosa* infections.

## DISCOVERY OF THE EXLB-EXLA TWO-PARTNER SYSTEM IN *P. AERUGINOSA* CLADES 3 AND 5

ExlA was first reported in 2014 by Elsen et al. during research on the hypervirulent, T3SS-negative strain *P. aeruginosa* CLJ1 (21). This strain was isolated from a patient with chronic obstructive pulmonary disease (COPD) associated with hemorrhagic pneumonia at the Grenoble University Hospital in France. Through comparative proteomics, a novel hemolysin in CLJ1 culture supernatant was identified and named ExlA. This toxin is secreted by bacterial cells and induces necrotic death in eukaryotic cells by forming pores in the host cell membrane (21).

ExlA is encoded within the *exlBA* operon, which consists of two genes: *exlB* (locus PA7_4641) and *exlA* (locus PA7_4642). The *exlB* gene encodes ExlB, an outer membrane protein required for ExlA secretion. In *exlB* mutant strains, ExlA is not detected in the bacterial supernatant, and the bacteria show no cytotoxicity toward the epithelial lung cell line A549 (22). ExlB is a 60 kDa protein containing two polypeptide-associated transport (POTRA) domains. The first domain (POTRA1) spans amino acids 85–160, while the second (POTRA2) is located between amino acids 162 and 230. Both domains are essential for ExlA secretion and cytotoxic activity (23).

Genomic analyses have shown that ExlA-positive strains are relatively rare within the *P. aeruginosa* population. A BLASTP analysis using the ExlA sequence from the PA7 strain as a reference identified 81 ExlA-positive genomes among 7,982 *P. aeruginosa* sequences deposited in the database (https://www.pseudomonas.com; consultation date: September 22, 2024), corresponding to approximately 1.01% of the analyzed strains. Similarly, Medina-Rojas et al. identified 33 *exlBA*-positive strains among 2,439 whole-genome sequences of clinical multidrug-resistant (MDR) *P. aeruginosa* isolates, representing 1.5% of the cohort (24). ExlA-positive strains have also been isolated from fecal samples of healthy animals (19).

These ExlA-producing strains are predominantly associated with *P. aeruginosa* clades 3 and 5. Early phylogenetic classifications divided *P. aeruginosa* into three major clades, represented by the reference strains PAO1 (clade 1), PA14 (clade 2), and PA7 (clade 3) (22, 25, 26). Clade 3 strains were considered phylogenetically distant “outliers” and were proposed by Rudra et al. as a potential new species, *Pseudomonas paraeruginosa* (27). One defining feature of clade 3 strains is the absence of T3SS genes and *rhlC*, which encodes rhamnosyltransferase 2, the enzyme responsible for adding a second rhamnose moiety to mono-rhamnolipids to generate di-rhamnolipids (28).

More recent genomic studies expanded this classification to five clades, with clades 4 and 5 being identified later (21, 29). Clade 4 was first reported by Freschi et al. in 2019 (29), although its low prevalence has limited detection in other studies (30, 31). For this reason, the present review focuses on clades 1, 2, 3, and 5. Notably, clades 3 and 5 lack T3SS genes and instead rely on the ExlA toxin as a major virulence determinant (21, 23).

Functional studies have revealed differences in virulence between ExlA-positive strains from these clades. Reboud et al. showed that clade 5 strains exhibit reduced cytotoxicity compared with clade 3 strains, despite harboring *exlA*. This reduced virulence was attributed to impaired exolysin secretion in clade 5 strains (17). Importantly, several clade 5 strains were isolated from clinical samples, indicating that isolation alone does not equate to high virulence. Instead, pathogenic potential depends on both bacterial factors and host conditions.

## MECHANISMS OF EXLA-MEDIATED DAMAGE IN EUKARYOTIC CELLS

Initial investigations on ExlA revealed its capability to create pores within host membranes, leading to the necrotic death of eukaryotic cells (21, 23). Basso et al. characterized ExlA as a protein comprising five β-sheet domains (Fil HAEM) and a hemagglutinin domain (HAEMA) that is conserved in filamentous hemagglutinin adhesins (FHA). Additionally, ExlA possesses five arginine-glycine-aspartic acid (RGD) motifs that are commonly involved in host cell recognition, although they do not contribute to ExlA cytotoxicity (23).

Notably, the ExlA C-terminal region, consisting of approximately 300 amino acids, plays a vital role in the hemolytic activity of epithelial A549 cells and is also important for bilayer recognition *in vitro* (23, 32). Further investigations employing cytoprotectants and cytolytic assays using red blood cells have demonstrated that ExlA acts as a pore-forming toxin, forming pores with a diameter of approximately 1.6 nm within host cells (23). Liposome assays established that ExlA requires phosphatidylserine to induce liposome leakage and that the C-terminal domain of ExlA can interact with negatively charged lipids found in eukaryotic cell membranes (32).

Effective delivery of the ExlA toxin requires close physical contact between *P. aeruginosa* and host cells. Unlike T3SS-dependent toxins, ExlA is secreted into the extracellular milieu and displays limited solubility, making its activity highly dependent on bacterial proximity to the target cell membrane. In this context, type IV pili (T4P) play a crucial role by mediating tight adhesion between the bacterium and host cells. T4P-dependent interactions allow ExlA-producing strains to maintain intimate contact with epithelial and immune cells, thereby facilitating efficient toxin delivery and subsequent pore formation in host membranes (23). This contact-dependent mechanism represents a key feature of ExlA-mediated cytotoxicity and distinguishes it from classical needle-based secretion systems.

**Fig 1:**
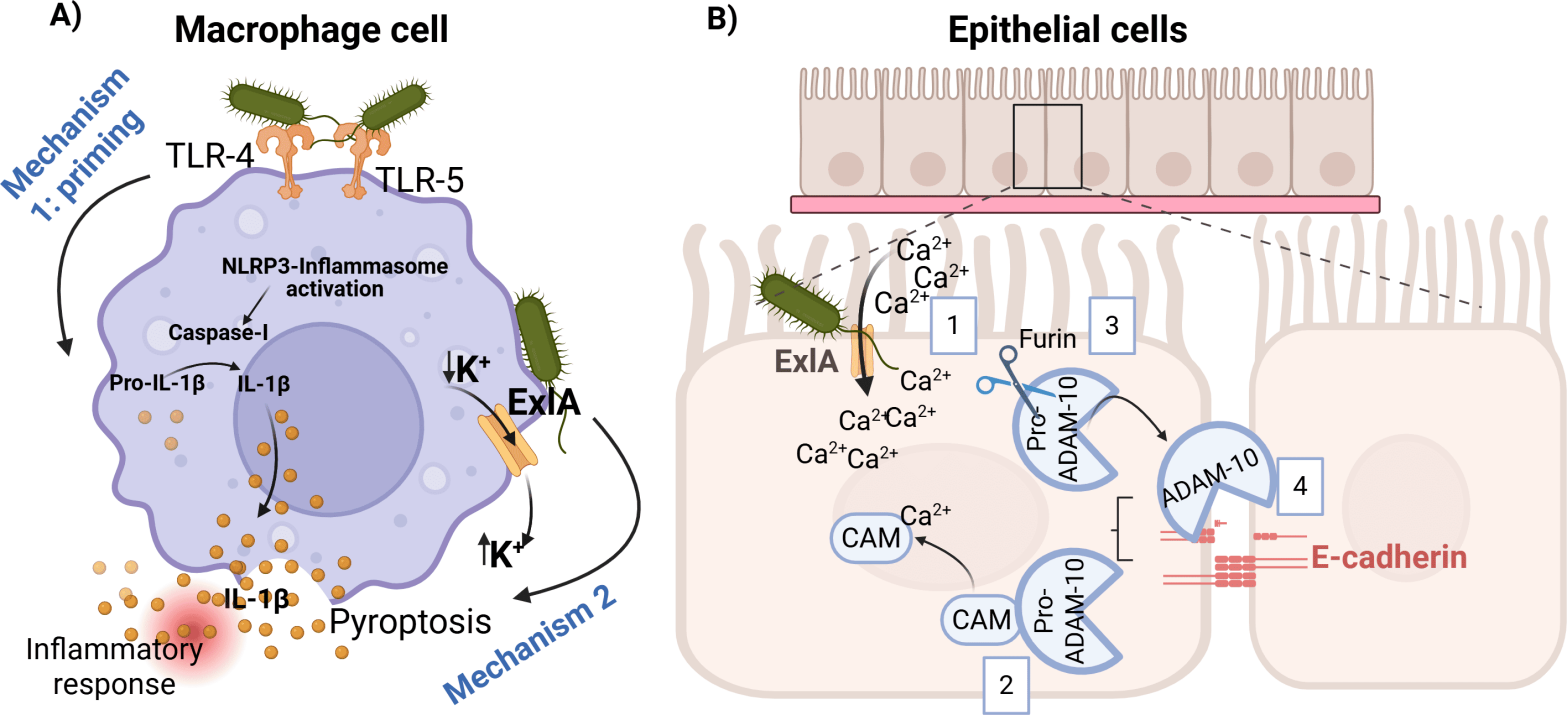
Mechanisms of ExlA-mediated inflammasome activation and epithelial barrier disruption. (**A**) In macrophages, ExlA induces inflammasome activation through two sequential mechanisms. The first is a process called priming signal initiated by recognition of LPS and flagellin by the transmembrane receptors TLR4 and TLR5, respectively, leading to NF-κB activation and transcription of pro-inflammatory genes, including pro–interleukin-1β (pro–IL-1β). The second mechanism is triggered by ExlA pore formation at the plasma membrane, causing K^+^ efflux, which promotes NLRP3 inflammasome assembly, caspase-1 activation, maturation of IL-1β, and pyroptotic cell death, resulting in IL-1β release. (**B**) In epithelial and endothelial cells, ExlA pore formation induces Ca²^+^ influx into the cytosol (1). Elevated intracellular Ca²^+^ binds to calmodulin, leading to its dissociation from pro-ADAM10 (2). Pro-ADAM10 is subsequently cleaved and activated by furin (3) and transported to the plasma membrane, where active ADAM10 cleaves cadherins (4), disrupting intercellular junctions in a T4P-independent manner. Figure created with BioRender.com.

In addition, *in vitro* experiments have shown that ExlA induces inflammasome activation via two mechanisms. The first consists of the process called “priming” that begins with the recognition of the LPS and the flagellum by the macrophage transmembrane receptors TLR4 and TLR5, respectively. Subsequently, the cell turns on the expression of genes encoding pro-inflammatory cytokines, such as pro-interleukin-1β (Fig. 1A) (33).

The second mechanism corresponds to inflammasome activation driven by ExlA pore formation, which causes a massive efflux of intracellular K^+^, thereby triggering NLRP3 inflammasome assembly, caspase-1 activation, maturation of IL-1β, and pyroptotic cell death, resulting in the release of pro-inflammatory IL-1β (Fig. 1A) (21, 33, 34).

In epithelial and endothelial cells, ExlA-induced pore formation triggers Ca²^+^ influx into the cytosol. Once inside the cell, Ca²^+^ binds to calmodulin, leading to its dissociation from the pro-ADAM10 metalloprotease. Pro-ADAM10 is subsequently activated by furin and transported to the plasma membrane, where ADAM10 cleaves cadherins and disrupts intercellular junctions in a T4P-independent manner (Fig. 1B) (35).

Therefore, the mechanisms of ExlA action found *in vitro* and in cell lines could explain hemorrhagic pneumonia and lung cell damage in patients infected with the previously mentioned CLJ1 clade 3 strain.

## REGULATION OF EXLA IN *P. AERUGINOSA*

**Fig 2:**
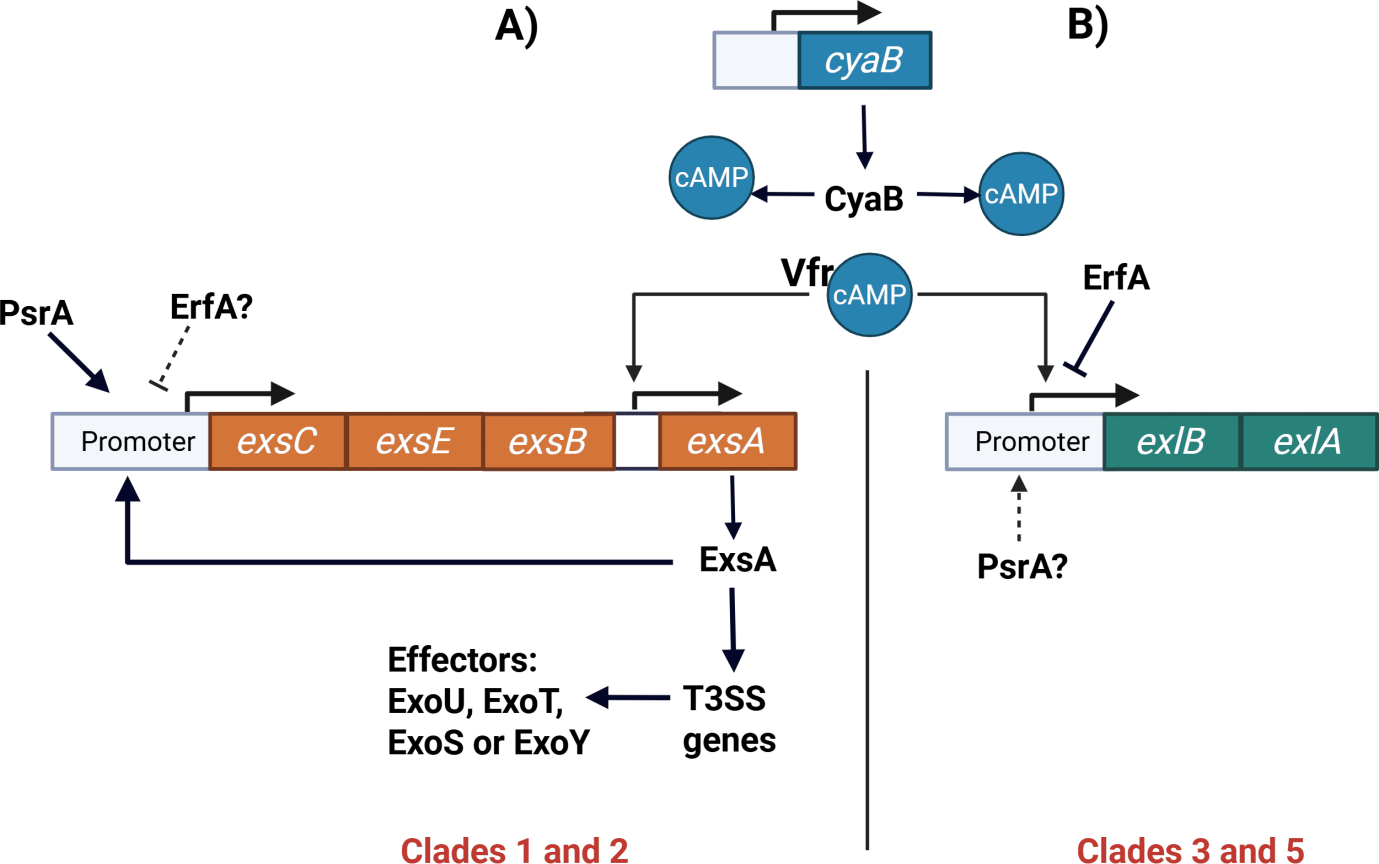
Regulation of T3SS and ExlA exotoxin expression by Vfr, cAMP, and ErfA in different *P. aeruginosa* clades. (**A**) In T3SS-positive *P. aeruginosa* strains belonging to clades 1 and 2, intracellular cAMP produced mainly by the adenylate cyclase CyaB activates the global regulator Vfr. Vfr directly stimulates transcription of *exsA* through an internal promoter within the *exsCEBA* operon. ExsA, in turn, acts as the master activator of T3SS gene expression, leading to the production and secretion of T3SS effectors, such as ExoU, ExoT, ExoS, or ExoY. The *exsCEBA* operon is also positively regulated by PsrA. (**B**) In ExlA-positive strains belonging to clades 3 and 5, Vfr–cAMP positively regulates transcription of the *exlBA* operon, which encodes the ExlA toxin and its outer membrane transporter ExlB. Expression of *exlBA* is negatively regulated by the transcriptional repressor ErfA, which binds to the *exlBA* promoter. The potential involvement of PsrA in *exlBA* regulation is indicated but remains to be experimentally confirmed. Together, these panels illustrate how Vfr and cAMP act as central regulatory hubs coordinating alternative virulence strategies in different *P. aeruginosa* clades. Figure created with BioRender.com.

Expression of the *exlBA* operon is tightly regulated by at least two transcription factors. One of these factors is the Vfr protein (Virulence factor regulator), which interacts with cyclic adenosine monophosphate (cAMP) to positively regulate the transcription of *exlBA* (Fig. 2). Accordingly, *exlBA* transcription is significantly reduced in a *vfr* mutant (36, 37). Increased intracellular cAMP levels enhance *exlBA* expression by promoting Vfr binding and transcriptional activity at the *exlBA* promoter. In *P. aeruginosa*, cAMP is synthesized by two adenylate cyclases, CyaA and CyaB, with CyaB acting as the primary source of cAMP under most conditions (38).

In addition to positive regulation, *exlBA* expression is negatively controlled by Exolysin Regulatory Factor A (ErfA). ErfA belongs to a conserved family of transcriptional regulators containing an XRE-like DNA-binding domain in the N-terminal region and binds the *exlBA* promoter as a homodimer (ErfA binding site: ACAACACTTTGTGTCAA) (Fig. 2). The C-terminal region of ErfA harbors a putative sensor domain with a cupin fold, suggesting that ErfA may respond to an as-yet unidentified signaling molecule. Functional studies demonstrated that disruption of *erfA* leads to derepression of *exlBA*, converting the originally non-cytotoxic *P. aeruginosa* strain PA70 into a cytotoxic phenotype due to ExlA overproduction (30, 39). These findings highlight ErfA as a key negative regulator of ExlA expression.

Despite the identification of Vfr, cAMP, and ErfA as central regulatory elements, several aspects of *exlBA* regulation remain unresolved. In particular, the signaling cues sensed by ErfA and the environmental conditions that fine-tune ExlA expression are still unknown, underscoring the complexity of ExlA regulation in *P. aeruginosa*.

## THE RELATIONSHIP BETWEEN THE T3SS AND THE EXLA SYSTEM

ExlA and T3SS systems are mutually exclusive virulence strategies in *P. aeruginosa*. To date, no strain has been identified that harbors both systems simultaneously, suggesting strong evolutionary or physiological constraints. One potential explanation for this exclusivity is the substantial energetic cost associated with maintaining and expressing two secretion systems with overlapping cytotoxic functions.

The evolutionary origin of this exclusivity raises an important question: how did *P. aeruginosa* acquire the *exlBA* operon while losing the T3SS? One possible explanation is that *exlBA* was acquired through horizontal gene transfer (HGT) between different *Pseudomonas* species. Supporting this hypothesis, several environmental *Pseudomonas* species, including *P. chlororaphis*, *P. fluorescens*, and *P. putida*, possess homologous *exlBA* operons, and ExlA-like toxins can be detected in these organisms. Notably, regulation of *exlBA* differs among *Pseudomonas* species; a Vfr-binding site within the *exlBA* promoter is unique to *P. aeruginosa*, indicating species-specific regulatory adaptation.

Other transcriptional factors that have recognition sites in the *exlBA* operon are MqsA in *P. putida*, NtrC and Mic in *P. fluorescens*, and GalR and FhlA in *P. chlororaphis*, suggesting that this operon is regulated differently between different *Pseudomonas* species, perhaps according to the survival needs and interacting organisms of each species (40).

In both clade 3 and clade 5 *P. aeruginosa* strains, the *exlBA* operon occupies the same chromosomal locus and remains under Vfr control (31). These observations suggest that a clade 3 ancestor initially acquired *exlBA* from other *Pseudomonas* species. In contrast, clade 5 strains likely originated from a clade 1 or clade 2 lineage that subsequently obtained *exlBA* through horizontal gene transfer from clade 3 strains. Additional evidence for evolutionary remodeling is provided by the presence of residual T3SS-associated genes in some ExlA-positive genomes, suggesting that loss of the T3SS occurred after acquisition of *exlBA* (31). However, alternative evolutionary scenarios cannot be excluded, including neutral gene loss, niche-specific selective pressures, or host-dependent adaptation.

HGT is a natural mechanism that contributes to the evolution of bacteria and consists of the transfer and acceptance of a gene or a set of genes, facilitating the ability of the receptor bacteria to adapt to specific environments. It has been proposed that *P. aeruginosa* loses the T3SS after acquiring *exlBA* genes because traces/scars of T3SS genes could be detected in the *P. aeruginosa* genome (31).

The elimination of the T3SS may reflect selective pressure to reduce the energetic burden of maintaining two secretion systems with similar anti-host activities.

Beyond these evolutionary considerations, the mutual exclusivity of ExlA and T3SS is also reflected at the regulatory level. In T3SS-positive strains, such as PAO1 and PA14 (clades 1 and 2), T3SS gene expression is controlled by the transcriptional activator ExsA, encoded within the *exsCEBA* operon (41, 42) (Fig. 2A). The *exsCEBA* promoter is positively regulated by PsrA, and an internal promoter upstream of *exsA* is directly activated by Vfr through a conserved binding site (43, 44). Similarly, in ExlA-positive strains, *exlBA* transcription is positively regulated by Vfr, highlighting Vfr as a central regulator coordinating alternative virulence strategies depending on strain background.

In contrast to this shared positive regulation, negative regulation of *exlBA* is mediated by Exolysin Regulatory Factor A (ErfA), a transcriptional repressor that binds the *exlBA* promoter as a homodimer via an XRE-like DNA-binding domain (Fig. 2B). ErfA contains a C-terminal cupin-fold sensor domain, suggesting responsiveness to an unidentified signaling molecule. Functional studies demonstrated that mutation of *erfA* converts the non-cytotoxic strain PA70 into a cytotoxic phenotype due to ExlA overproduction (39).

ErfA is conserved in the clade 1 reference strain PAO1; however, whether it also participates in the regulation of the *exsCEBA* operon remains unknown.

Together, these findings indicate that while ExlA and T3SS are evolutionarily and functionally exclusive, they remain connected through shared regulatory elements, particularly Vfr and cAMP signaling (Fig. 2). Although significant progress has been made in identifying key regulators of the *exlBA* operon, many aspects of ExlA regulation remain unresolved. In particular, the potential involvement of PsrA in ExlA regulation and the nature of the signal sensed by the ErfA sensor domain remain open questions. Addressing these gaps will be critical for understanding how *P. aeruginosa* coordinates virulence expression and adapts to diverse host environments and may provide insights relevant to antivirulence strategies.

## BIOTECHNOLOGICAL POTENTIAL OF NON-VIRULENT *P. AERUGINOSA* CLADES 3 AND 5 STRAINS

As mentioned previously, both strains belonging to *P. aeruginosa* clades 3 and 5 possess the *exlBA* operon. However, some strains from both clades displayed attenuated or non-virulent phenotypes (17, 45, 46). One explanation for this is that these strains overexpress the negative regulator ErfA, which suppresses *exlA* expression. Another possibility is that they harbor mutations in genes responsible for synthesizing key proteins required for *exlA* expression, as observed in strain PA7. The clade 3 type strain PA7 carries a non-functional *vfr* due to a frameshift mutation, leading to reduced cytotoxicity due to diminished *exlA* expression (36). Additionally, they lack T3SS, which is a crucial virulence mechanism in *P. aeruginosa* (47, 48).

This opens up the possibility of using these non-virulent strains for biotechnological applications. Recently, the use of clade 5 strains for biotechnological applications has been explored (49), although it has previously been considered for clade 3 strains. For example, evidence has shown that the attenuated virulence of *P. aeruginosa* ATCC 9027 is multifactorial. Although this strain carries a defective *lasR* gene that disrupts quorum-sensing regulation, overexpression of *vfr* or *cyaB,* which increases intracellular cAMP levels and activates the *exlBA* operon, failed to restore virulence in murine infection models (37). These findings indicated that the non-virulent phenotype of ATCC 9027 is stable and not easily reverted, reinforcing its potential as a safe host for industrial rhamnolipid production. Another example is strain CR1, which, like the ATCC 9027 strain, does not have T3SS, but can produce secondary metabolites and is still non-virulent in animal models (45, 46).

*P. aeruginosa* can synthesize a large arsenal of secondary metabolites, such as pyocyanin, which can be used as an antifungal compound in agriculture (13, 50), and rhamnolipids, which can solubilize compounds and can be used in cosmetology and environmental bioremediation (12, 51). Proteases, lipases, and chitinases can be used to obtain chitin from shrimp waste (14, 52, 53). To date, it has been difficult to produce these products on an industrial scale, as well as to commercialize them because they are produced by potentially virulent strains. Utilizing opportunistic pathogens in biotechnology entails various risks, such as health hazards stemming from potential infections, environmental contamination if containment measures are inadequate, dissemination of antibiotic resistance, unforeseen outcomes of genetic alterations, and regulatory concerns necessitating rigorous safety protocols and oversight to minimize adverse effects on public health and the environment. To address these risks, several attempts have been made to obtain attenuated strains. For example, Grosjean et al. (54) modified the genome of *P. aeruginosa* PAO1 (clade 1), resulting in SM54 strain, to create a safe platform for biotechnological applications. Although the virulence tests conducted with SM54 demonstrated a decrease in mortality rates in both CD1 mice and *G. mellonella* larvae models compared to those infected with the PAO1 strain, animals infected with the SM54 strain continued to succumb to infection, albeit at a slower rate than those infected with the PAO1 wild-type strain. The authors noted the necessity of further eliminating the genes associated with the virulence of the SM54 strain to reduce its pathogenicity. However, they also acknowledged the potential impact of these mutations on bacterial physiology and stressed the need for additional studies to achieve the desired product performance (54).

Additionally, mutations were carried out at key points in reference strains of *P. aeruginosa* to obtain a safe platform for synthesizing metabolites of biotechnological interest (54). Genomic reduction has the potential to enhance the synthesis of value-added products. However, their current drawback is the time-intensive nature of their construction methods. By enhancing existing genome editing tools and developing new ones, along with leveraging *in silico* models, these strategies could be integrated to accelerate the process, leading to the creation of more efficient and streamlined cell factories (55).

Owing to the studies carried out on these non-virulent clade 5 strains, it is possible to have more options for the exploitation and application of secondary metabolites from this fascinating bacterium.

The use of non-virulent *P. aeruginosa* to produce metabolites has key advantages. For example, exceptional metabolic versatility can be maintained. This broad adaptability makes it an ideal organism for the industrial-scale production of these biomolecules. In addition, it possesses well-characterized metabolite biosynthetic pathways that simplify genetic manipulation to optimize industrial production. Established growth processes for *P. aeruginosa* have already been optimized for the efficient and scalable production of these biomolecules. While other bacteria may produce lower amounts of the same compounds, they often require more specialized and expensive growth media or have slower growth rates, which increases the production costs. The genetic tools available for engineering *P. aeruginosa* are also well developed, allowing easy modification to enhance production yields or reduce virulence, which may be more difficult or less reliable in alternative bacterial models.

## LIMITATIONS OF ALTERNATIVE BACTERIAL HOSTS FOR RHAMNOLIPID AND PYOCYANIN PRODUCTION

The most extensively studied and exploited metabolites of *P. aeruginosa* are rhamnolipids, which have attracted considerable attention because of their biotechnological potential and are now commercially available (56). Efforts have been made to produce this molecule in different bacterial hosts, but the first consideration for heterologous production is the selection of bacteria that can synthesize rhamnolipids without harm during the process. Gram-positive bacteria are unsuitable for this purpose because rhamnolipids exhibit strong antimicrobial activity against them. The single cytoplasmic membrane of Gram-positive bacteria, encased in a peptidoglycan layer, does not provide an effective barrier against rhamnolipids (57).

In contrast, Gram-negative bacteria can be used to synthesize rhamnolipids. For example, *P. putida* KT2440 pSynPro8oT_rhlAB and *E. coli* BL21, using glucose as the single carbon source, produced a rhamnolipid concentration of 14.9 g/L (58) and 0.64 g/L (59), respectively. None of the heterologous rhamnolipid producers has yet reached levels of at least 36.7–40 g/L that can be obtained with *P. aeruginosa* (56, 60). Recently, *P. chlororaphis* subsp. *chlororaphis* ATCC 9446 was tested to produce rhamnolipids, producing only 87% of the amount produced by *P. aeruginosa* PAO1 (61). Although various genetic modifications have been reported to improve rhamnolipid production using *P. putida* KT2440 as a heterologous host, this platform has several limitations (62). The goal is to match or surpass the rhamnolipid production levels achieved by *P. aeruginosa,* which can be achieved using non-virulent *P. aeruginosa* strains.

Thus, research should continue to explore rhamnolipid production in other bacterial models and improve the conditions needed to achieve this. However, the production conditions for *P. aeruginosa* have already been well established. The main concern of the industry is the virulence of *P. aeruginosa*. With the identification of non-virulent strains, such as some within clade 5, this issue has been largely mitigated. In contrast, using alternative bacterial species entails higher production costs owing to their slower growth, the need for costly substrates, and stricter environmental requirements.

Additionally, efforts have been made to produce pyocyanin in *E. coli*, achieving a yield of 18 mg/L (63) compared to the 33 mg/L produced by *P. aeruginosa* OG1 (64). However, producing this secondary metabolite in *E. coli* is challenging because this bacterium struggles to cope with the reactive oxygen species (ROS) generated by pyocyanin, which is toxic to the cell (65). Attempts to boost *E. coli* defense mechanisms by overexpressing antioxidant enzymes such as superoxide dismutase and catalase have proven ineffective in improving its tolerance to pyocyanin. Combining these enzymes or overexpressing the oxidative stress response regulator, SoxS, did not increase *E. coli* resistance, making it difficult to produce pyocyanin in this host (63).

The exploration of *P. aeruginosa* clades 3 and 5 offers promising opportunities for biotechnological applications because of the non-virulent nature of some of these strains (17, 45, 46). Furthermore, non-virulent isolates can safely produce valuable metabolites, such as rhamnolipids and pyocyanin, based on their intrinsic metabolic routes and resistance mechanisms, and can be genetically modified to produce higher levels.

The well-established growth conditions and biosynthetic pathways of *P. aeruginosa* make it a viable option for industrial applications. Nonetheless, the development of non-virulent strains and advancements in genome editing technologies could enhance the safe and efficient production of these metabolites, positioning three and five non-virulent strains as significant contributors to sustainable biotechnological processes.

## FUTURE DIRECTIONS

Currently, research on *P. aeruginosa* strains belonging to clades 3 and 5 is limited, as most efforts aimed at developing new therapies have focused primarily on strains from clades 1 and 2. Although it is well documented that clade 3 strains, such as the hemorrhagic pneumonia isolate CLJ1 and the human urinary tract isolate *P. aeruginosa* IHMA879472, can cause severe infections through secretion of the ExlA toxin, the potential for treating infections caused by clade 3 strains remains underexplored, highlighting the need for further investigation in this area. Clade 5 strains, such as *P. aeruginosa* PA70, generally display attenuated cytotoxicity.

It is essential to evaluate whether proposed alternative anti-pseudomonal therapies can effectively inhibit the pathogenicity of these relatively understudied phylogenetic groups or whether vaccines targeting conserved factors across all clades can be developed. Additionally, investigating the fitness costs associated with strains harboring both T3SS and ExlA could provide valuable insights into the evolutionary pressures driving T3SS loss. Further identification and characterization of clade 5 strains are necessary to better define their pathogenic potential.

Moreover, the development of methods to accurately identify clinical isolates and generate epidemiological data is crucial for assessing the incidence of infections caused by different clades, thereby enabling appropriate and timely treatment strategies. Comprehensive phenotypic characterization of strains with the potential for secondary metabolite production, despite their reduced pathogenicity, is also important for biotechnological applications. Notably, clade 5 strains studied to date exhibit attenuated cytotoxicity in several cell lines, reduced virulence in *Galleria mellonella* larvae, and possess the genetic machinery required for the biosynthesis of secondary metabolites of biotechnological interest. Consequently, clade 5 strains represent a promising resource for future biotechnological applications.
